# From dumping to circular economy: There is no success like failure

**DOI:** 10.1177/0734242X231221084

**Published:** 2024-01-19

**Authors:** Håkan Rylander, Anders Lagerkvist

**Affiliations:** 1 H Rylander Management AB, Vellinge, Sweden; 2 Luleå University of Technology, Luleå, Sweden

**Keywords:** Waste, management, development, history, research, legislation, technology

## Abstract

Waste management has been developing in response to needs. The need to get rid of unwanted materials has always been a motivation but using the resource value of waste has also been a driver from the stone age and forwards. In affluent times not so much. Sanitation became a motivation with the discovery of pathogenic microorganisms in the mid-19th century, and after World War 2 (WW2) a strong focus on environmental protection developed, and in recent times, the resource aspect has received an interest despite material affluence. Legislation has been one of the drivers for recent developments, in the case of Sweden, the environmental protection legislation came in the late 1960s, and a few years later, the municipalities got the exclusive right to collect and manage household waste. Many local and regional waste management companies were established, owned by the municipalities. These organizations became agents of development, due to the increased scope and capacity. Adding to the environmental protection agenda, a renewed interest in waste as a resource was initiated by the oil crises of the 1970s, resulting in new waste incineration plants, with energy recovery, connected to already existing district heating networks. Mistakes, failures and alarms in the 1970s and the 1980s resulted in treatment method improvements and the establishment of source separation as an integral part of waste management. The waste management community stands strong today and is taking a more proactive role than before, which includes a stronger focus on communication with other stakeholders.

## Introduction

This text deals with the development of waste management with a focus on the territory of Sweden. Neither of the authors is a professional historian and the text is just representing their perspectives, experiences and knowledge. *Håkan Rylander* has served as MD/CEO of a national association and of two regional waste management companies and is a past president of International Solid Waste Association (ISWA). *Anders Lagerkvist* has developed Waste Science and Technology as an academic discipline in Sweden and is also a founding member and past president of International Waste Working Group (IWWG). Together, they have almost a century of working with waste management in theory and practice, and the aim of this article is to use this familiarity with the area to bring forward a readable story that can be of help to those who want to understand what is and has been, driving the development of waste management.

In the first section, the development in older times is presented and interpreted by the different interests that have emerged over time, for example, the resource-, hygiene- and environmental protection interests.

In the second section, a timeline of some failures, alarms, and, not least, successes that have been milestones in the development of municipal waste management is presented.

In the following section, a timeline for the coupled development of academic waste research is presented with some observations about the quantity and theme of theses – without any claim of completeness or objectivity.

In the final section, a condensed view is presented of what the challenges for waste management development have been, are and will be.

## Emerging waste management in older times

The early hunters/collectors, who came to our country when the inland ice melted away, have not left many traces of themselves except a few tools like heads of arrows and spears. With the more stationary farmer cultures during the Stone Age, the remains begin to be more notable. From the later Swedish Bronze Age (from about 1000 BC), there are many remains around Lake Mälaren that can be interpreted as small refuse dumps. These consist of stone fragments from boiling stones and hearths, mixed with other refuse such as soot, ash and food leftovers. Some piles seem to have originated under a hearth that was used for a long period of time and others seem to have been thrown beside a settlement. Some of the piles also seem to have been used as tombs (Hyenestrand, 1967).

A couple of millennia later, in the trading cities of the Vikings, the waste accumulation grows with urbanization. A well-known example is the so-called Black Earth on Björkön in Lake Mälaren where the city Birka was situated. Birka was a trading city, which during its time of prosperity in the 9th and 10th centuries is believed to have had a population of about 2000. The Black Earth, soil mixed with ash, coal and other waste products, is a 1–2.5 m thick layer that covers an area of about 6 ha (Nordisk Familjebok, 1905). The concentration of material flows that Birka, and other trading places generated do not seem to have induced more organized waste disposal, the waste accumulated in and around houses, filled pits and marshes, etc. However, the problem of waste accumulation was acknowledged early on in rules and laws, for example, concerning the order in which the streets should be swept (starting at the high end and sweeping down).

The layers of waste, which can be found in the foundations of the ever-larger cities developing during the early Middle Ages, seem to have grown with the economy of the times – in good times more waste was generated and in poor times, the materials were used more economically. In the typical strata of a northern medieval city, one also finds layers of ashes and building materials between the waste layers, following the periodical fires and rebuilding of these wooden house cities.

The trade routes, by boat during the summer and by sled during the winter, determined the siting of cities to where the trade routes crossed and the defence possibilities were good. Cities then expanded into the adjacent water, which was often shallowed by waste. In Stockholm, for instance, the area of Gamla Stan (Old Town) became three times larger in the 18th century compared to the 13th century, despite its steep shores. Filling depths of up to about 20 m of mixed waste, ashes and construction wastes have been found at excavations in Stockholm (Hansson, 1970).

Separate sites to which unwanted material was transported did not seem to appear until the later part of the 19th century. At that time, the interest in hygiene resulting from the discovery that cholera was spread via waterborne microorganisms led to the creation of municipal waste management organizations. These organizations were often designed in a military fashion, presumable to give waste management a status lift. Before this, waste was mostly looked upon as just a practical material handling problem. Source separation and recycling of useful waste were practised when needed – otherwise, the waste was dumped in the most convenient way. The work to keep public places clean was assigned to socially low-ranking people with whom ‘honest’ people would associate as little as possible.

**Figure 1.**
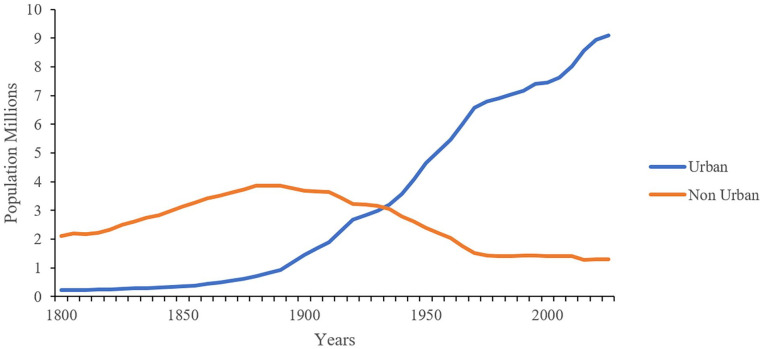
Swedish population distribution between urban and not urban areas between 1800 and 2020. Data from SCB (2021).

Sweden was a mainly agricultural country up until the middle of the 19th century, when an industrial expansion began, starting with the sawmill industry. With the expanding industrialization and urbanization, the country rapidly changed into one where most of the population resided in towns and cities, see Figure 1. This led to a further concentration of material flows and the resulting waste problems forced the development of new solutions. In the cities, waterworks were built, and then, at the turn of the century, wastewater pipes made it possible for a new convenience to become widespread – the water closet. In addition, special sites were created for the accumulation of such waste that could not be reused, although dumping in water was still common, as was open burning.

Towards the turn of the century, it becomes more and more common for cities to have special entrepreneurs or municipal organizations handling refuse collection and disposal. Keeping the streets clean and collecting latrine are still the predominant chores, but the complexity of the waste streams grow at the same rate as new consumer products are coming into use.

In 1930s, the refuse chute was introduced by Hyresgästernas sparkasse - och byggnadsförening (HSB), a consumer co-operative building association. With the introduction of this convenience in multi-family buildings, the pattern of packaging mixed waste in a bag by the consumer was established.

**Figure 2.**
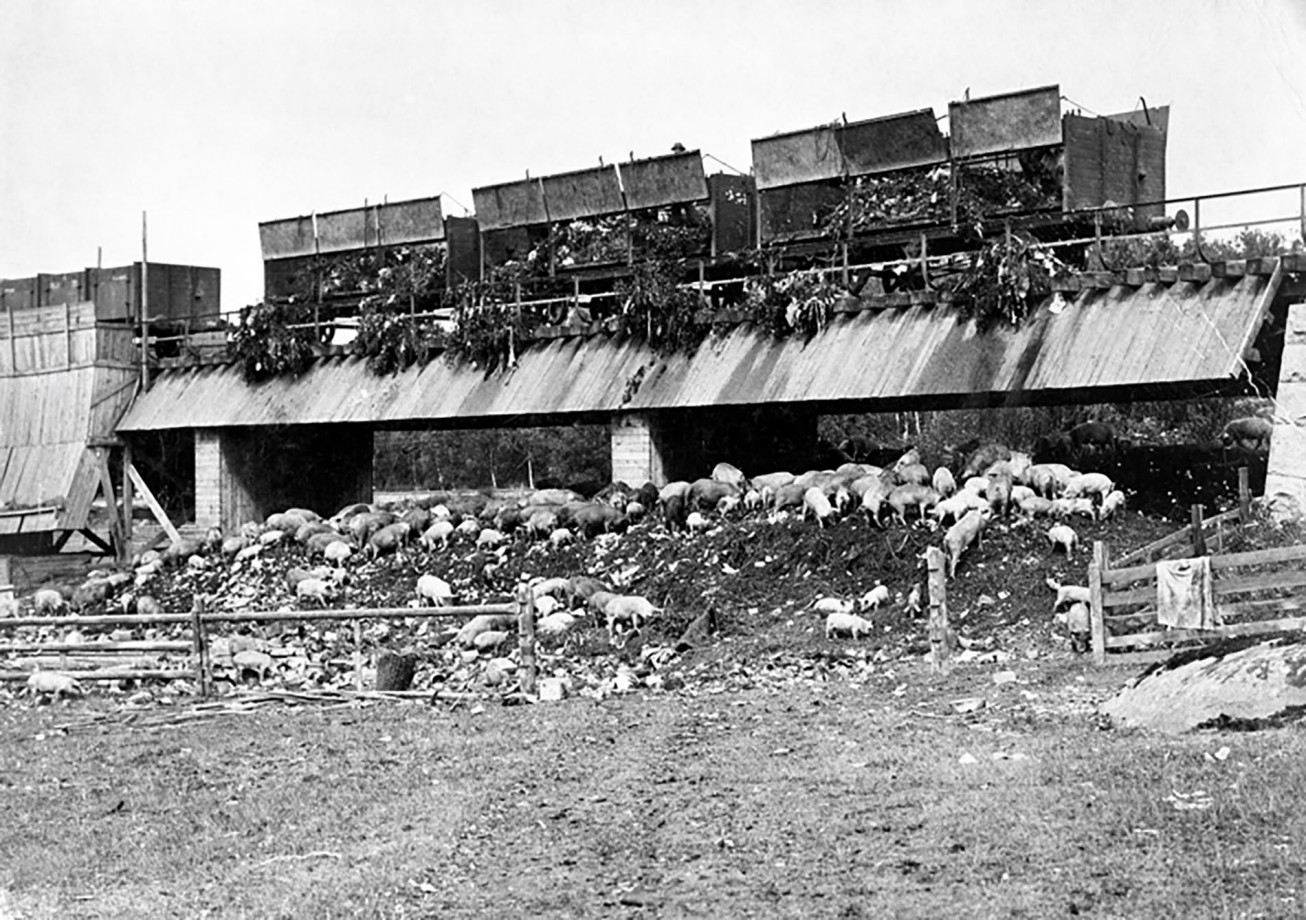
Kitchen waste delivery by railway at Lövsta recycling facility west of Stockholm in 1897 (Stockholms Stadsmuséum, 2016).

Waste management was adapting as well as possible to increasing volumes and new combinations of materials, but it became increasingly difficult to use waste in any reasonable way. Using the fertilizing contents of organic waste was no longer interesting with decreasing amounts of latrine and a growing percentage of non-compostable materials in the household waste, such as tin cans that became increasingly used for food packaging after World War 1 (WWI). As a consequence the use of food waste, as depicted in Figure 2, became increasingly difficult and facilities were closed down. The landfills grew in size and number. Due to faster filling rates, the emerging use of compactors, and the use of daily cover, the landfills/dumps turned predominantly anaerobic and that intensified leachate and odour emissions, which resulted in many negative reactions from neighbours. The growth of both the cities and the waste generation aggravated the problems, and as a result, new landfills were established further and further away from the cities, but the packaging of mixed waste prevailed and the lion’s share of Municipal Solid Waste (MSW) ended up in landfills. Up until the 1950s, the district heating systems were not sufficiently developed for waste incineration with energy utilization to be an interesting alternative – and after WW2, the decreasing price of oil was another factor dampening ambitions to use waste heat.

## Modern waste management emerge

### Growing awareness and new legislation

**Figure 3.**
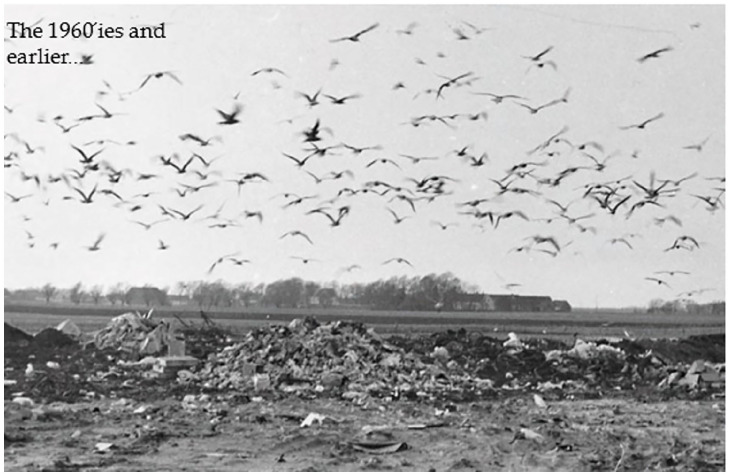
A dumpsite view from the 1960s.

Although many cities and municipalities in Sweden early had realized, mostly for hygienic reasons, the need of collecting household waste and delivering it in a restricted and controlled area, the overall situation at the beginning of the 1960s was far from satisfying. There was no control, with some exceptions, from the authorities of pollution, leachate, uncontrolled fires, etc. where the waste was dumped (Figure 3). Especially dumps with a hazardous waste content turned out as a very severe problem. There were a few waste incineration plants but with no or little use of the energy content in the waste. The collection of household waste was mostly done by private contractors, based upon an agreement between the contractors and the property owners. In some cities, for example in Stockholm, 5–6 contractors could turn up at different times of the day in a small street to collect household waste from different properties, without any coordination, causing noise, traffic jams and emissions from the vehicles. There was a slowly growing awareness that there had to be better control of the collection and handling of household waste.

That awareness about the increasing environmental problems, in general, grew even stronger with Rachel Carson’s book ‘Silent Spring’ (Carson, 1962) and the book written by the Swedish researcher Hans Palmstierna ‘Plunder, Starvation, Poisoning’ (Palmstierna, 1967). The publications of the books were followed by an engaging and intensive debate. The problems of increased littering all over Sweden and a common public mentality of ‘buying, wear, tear and throw away’ was also an important part of the debate.

As a result of the intensive debate and the increasing awareness of our environmental problems, the Swedish Government decided in 1967 of setting up the Swedish Environmental Protection Agency. In 1969, the Swedish Parliament enacted the first Environmental Protection Act in Sweden (SFS, 1969), coming in force the same year. The handling of waste was, from an environmental perspective, included in the new law. The more detailed handling of waste was later described in The Ordinance on Environmental Protection (SFS, 1981). There have been many changes in the Act since 1969, and in 1998 all environmental protection legislation was gathered in the Environmental Code (SFS, 1998), with appurtenant different ordinances. Legislation concerning waste management is since 1998 gathered in one chapter in the Code.

### The municipal waste management act

However, even if there now was legislation concerning the environmental aspects of waste, there was still a very confusing and unsatisfactory situation concerning the handling of household waste in Sweden, from an organizational, judicial and economic aspect. To give one judicial body control, the Swedish Parliament in 1972 decided upon the Municipal Waste Management Act (SFS, 1970), stating the legal/judicial and economic responsibility for the Swedish municipalities to collect and take care of household waste in an environmentally correct way.

### Development, failures and successes

The enforcement of the new legislation on municipal responsibility worked very well, with all Swedish municipalities now organizing the collection and final handling of household waste in accordance with the intention of the new law. The collection of household waste was still very much carried out by private contractors, but now with a contract between the municipality and one or two contractors for the collection throughout the municipality. In response to the enlarged municipal responsibility, many local and regional waste management companies were established, and owned by the municipalities. These organizations also became agents of development, due to the increased scope and capacity.

Besides organizing the collection of household waste, the focus from the municipalities was very much on the closing of old dumps, turning a few of them into landfills and the siting and establishment of new landfills. The main problem was how to deal with the leachate from the landfills in a correct way. Tests were done with different kinds of local treatment at the landfills as well as pumping the leachate to a municipal sewage treatment plant. During the 1970s, there was a belief that milling the household waste, after collection and before landfilling, would significantly reduce the needed volume for landfilling and speed up the degradation of the waste, especially if being mixed with sewage sludge. A large number of hammermills were sold on the Swedish market to be used not only for milling the waste before landfilling but also in the separation plants, which were built at the end of the 1970s, in the belief that it would be easier to separate and recycle the waste if it was mixed and crushed into small pieces.

All of it turned out to be a complete failure. The initial aerobic degradation that was reducing the leachate concentrations in the traditional landfill was shortened by the changes. Milling the waste opened up a larger specific area resulting in a quick consumption of available oxygen and the onset of intense anaerobic degradation, resulting in more polluted leachate, even harder to handle, especially if the milled waste had been mixed with sewage sludge. The increased use of compactors at the landfills aggravated the problem. The milled and mixed waste brought to the mechanical separation plants turned out to be completely impossible to separate into new recyclable products.

**Figure 4.**
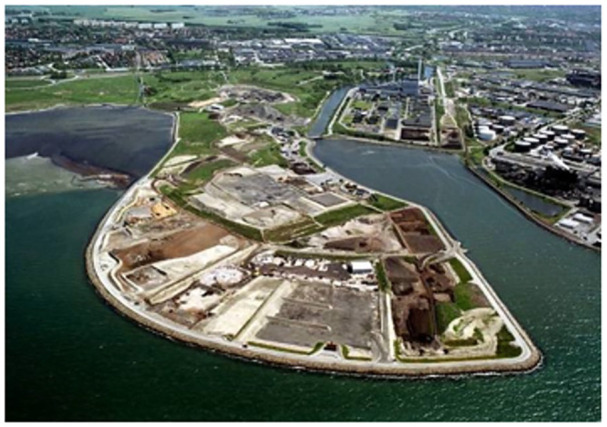
A modern landfill – a recycling site.

The very small part of the household waste that is landfilled today is not milled any longer and is not mixed with sewage sludge. All that stopped in the 1980s. But the problems motivated a lot of research and development that increased the competence of the industry with regard to what was going on in landfills and how the leachate could be managed (Figure 4).

### Improved working conditions

The refuse chute, introduced in the 1930s in new multi-family houses, was a very convenient and hygienic way of getting rid of waste for households. With this, the source separation of different waste in different bins ended. You did not have to go down into the cellar or out in the backyard to put the waste in bins. Another expected benefit of the new procedure was a reduced number of rats and other vermin accessing the food waste. Most of the installed chutes ended up in a bin in a small room in the cellar, with access only via steep and narrow stairs, which was a problem for those doing the collection of the heavy bins. The difficult working conditions were more and more discussed and put into focus. In 1976, there was a wild strike in Stockholm, the garbage collectors stopped working due to the very difficult and bad working conditions in the old town of Stockholm. The strike resulted in new and significantly improved and stricter directives for the design of the storing areas for waste in the multi-family houses and how to transport waste out of them. The Swedish Association of Waste Management (today Avfall Sverige, so also named in the following), which now had become a real national association for all those engaged within waste management, was very active with lobbyism and proposals in the preparations of the new directives, presented by a Government Agency. With time, more and more chutes were closed in order to improve the working conditions, and also to encourage and facilitate an increasing source separation of waste in the households. Source-separated fractions are today brought by households to different bins at collection points, recycling stations and recycling centres.

So, what started as improved hygienic conditions and convenience for the households ended with time in problems with the working conditions for those collecting the waste, in turn resulting in stricter and significantly improved working conditions and also with time in successful source separation of waste!

### A big failure and its consequences

In 1975, the Swedish Government presented a proposal ‘Recycling and disposal of waste’ (Governmental Proposal, 1975), accepted by the Parliament the same year, in which, among others, they presented promises of markets for all materials being sorted, separated and composted out of *mixed* household waste, without any pre-separation at the source! The promises were given without any pre-investigation, any review or any clarification of the pre-conditions for such existing markets! The idea of the Polluters Pay Principle was introduced, without any further concepts or measures.

Governmental grants were given to all new mechanical separation and composting plants. Between 50 and 100 million euros were given to 22 new plants all over Sweden, from 1976 to 1982. Separated materials and compost of a very bad quality were looking for a market, with no demand at all! Desperate plant owners tried in many ways to improve the quality of the separated fractions and of the produced compost it was not possible to produce satisfying qualities out of mixed household waste; in most cases, it was milled as a first step when entering the plants. The first notice of the problems came already in 1978 when Avfall Sverige carried out and published a study on behalf of the Nordic Council of Ministers (Nordiska Ministerrådet, 1978). The study was an overview of different methods for the treatment of household waste in the Nordic countries, including waste incineration, milling, separation/recycling, composting and pyrolysis (two pilot plants, one in Denmark and one in Sweden). Thirty different plants were included in the study. The study indicated that already in 1978 there were problems with the quality of the ‘recycled’ fractions at the separation plants as well as with the quality of the produced compost.

It turned out that pyrolysis did not work with such a heterogeneous fuel as household waste, not even on a pilot-plant scale, and that was even more confirmed later on when three commercial, full-scale plants were established in Germany, Luxemburg and France. Nor did pyrolysis work in commercial, full-scale with such a heterogeneous fuel at any of these plants. After these failures, there has not been any serious interest in the pyrolysis of waste in Sweden. It may work for very homogeneous fractions.

Based upon the severe problems with the market for the separated fractions and for the produced compost, Avfall Sverige tried to stop the governmental grants to even more mechanical separation and composting plants already at the end of the 1970s. The effort paid off, and at the beginning of the 1980s, a big study started, in cooperation between the Swedish Environment Protection Agency and Avfall Sverige. The aim was to evaluate the operational results, the markets for different products, the problems, the advantages, the disadvantages, the economic results and the working conditions at the Swedish separation/composting plants and also at the Swedish waste-to-energy (WtE) plants. Both treatment methods were evaluated to get a fair comparison and to develop advice and guidelines for future waste treatment in Sweden.

The study was continuously reported, with an extensive final report in 1986 (DRAV, 1986). The conclusion concerning the separation/composting plants was very much a confirmation of a number of problems which had been seen already at the beginning of the 1980s – big operational problems, very bad quality of the separated fractions and of the compost and the failure to market/to sell/to get rid of the separated fractions and the compost. All these problems of course also resulted in severe problems with the economy.

Due to the critique from the industry, governmental grants for new plants ceased already in 1982. At the end of the 1980s, all separation/composting plants were closed down or operating only with parts of their equipment. Composting of mixed household waste ceased completely. Garden waste was already collected separately and composted, and still is.

The very negative experiences from the mechanical separation of mixed household waste paved the way for a very successful concept, Source Separation and all the improvements following that.

### WtE, from fast development to threat to successful stability

At the beginning of the 1970s, there still were just a few waste incineration plants in Sweden, some of them even without energy recovery. The responsibility, by the law in 1972, for the municipalities to take care of household waste, combined with the first international oil crisis of 1973 resulted in substantial interest in establishing new waste incineration plants and now with energy recovery. The possibility to connect the plants, with their heat production, to the already existing district heating systems in all large and midsized cities in Sweden very strongly contributed to the fast establishment of a number of new WtE plants. The final report from the study between the Swedish EPA and Avfall Sverige, see above, stated that WtE was an established and functional method for handling waste, but with a need for continuous optimization, continuous maintenance, utilization of produced heat and electricity and an all the time very well-functioning flue gas cleaning, including cooling off and condensation with energy recovery from exhaust gases.

### Dioxins

The above-mentioned report had just been published when a real threat to WtE appeared – a dioxin alarm in 1985. Professor Rappe at Umeå University presented high emissions of polychlorinated dioxins from waste incineration plants not only in Sweden but also worldwide, resulting in an intensive and aggressive debate about the existence of waste incineration in Sweden (Rappe, 1984). The Swedish EPA declared in 1985 a moratorium on the construction of new waste incineration plants in Sweden. After negotiations and promises from the plant owners, all of them represented in a special working group within Avfall Sverige to meet all requirements and to install new and efficient gas cleaning technology at the Swedish waste incineration plants to significantly reduce the emissions of dioxins, the Swedish EPA cancelled the moratorium in 1986. It all resulted in a fast introduction of new gas cleaning technologies, with significantly reduced emissions of dioxins and a very much improved high-energy efficiency. The Swedish waste incineration plants became modern WtE plants, with high efficiency and with a minimum of hazardous gas emissions, and still are. Heat as well as power is produced and utilized, and only very little heat is cooled off.

### Methane gas collection

After impulses and experiences, especially from Germany, the collection of methane gas from the landfills started in the early 1980s, increasing all over Sweden during the following 1980s. The reason was of course to minimize the emissions of methane and take care of it in a safe way. To flare it away or even better by using it for power production. At its peak, the national landfill gas recovery was about 1 TWh per year or about 0.2% of the energy use in Sweden.

### Maximum recovery and recycling of material and energy and a minimum of landfilling

At the end of 1980s, there was an increased focus on the development and on the improvement of all steps within waste management – from landfilling and leachate to recycling and recovery of materials and energy. The discussions, ideas and concepts of production and living with almost no waste started. The activities at the universities stimulated politicians, authorities and those operations within waste management in Sweden. ‘When produced – waste a resource to be better used!’

Already at the end of the 1980s, the operational goal became: ‘Maximum recovery and recycling of material and energy and a minimum of landfilling’, achieved by a combination of methods, where different wastes are directed to an environmentally and resource correct handling – reuse, recovery, recycling, biological treatment, energy recovery and landfilling.

The ambitions to increase the recycling of different materials – the collection of glass, wastepaper, cardboard and other packaging – grew bigger at the end of the 1980s. Based on the negative experiences from the mechanical separation facilities, during the end of the 1970s and the 1980s, schemes including source separation were established. Source separation very quickly turned out to be successful as the separated materials were clean and not polluted by each other or by ordinary household waste. Also, the collection of household hazardous waste increased with separate collection of batteries, electronic scrap, etc., from the households. The results encouraged the politicians and waste management in Sweden to take further steps.

In 1990, 60% of the household waste was still landfilled without any other treatment, and 40% was incinerated with energy recovery and to a small extent, it was recycled.

### Municipal Waste Plans

n 1991, the Swedish Environmental Agency published a regulation on Municipal Waste Plans about the prevention and the management of waste. Every municipality is since 1991 responsible for establishing a 4-year plan of how to meet and fulfil governmental goals, directives and ordinances, and since Sweden joined EU how to meet with EU directives and regulations. The plan must be very flexible, with possibilities to make changes in it during the 4 years. Municipalities being owners of a regional waste management company, with very few exceptions, present a joint plan, which then will be approved upon in each municipality.

The plan has turned out to be a very efficient document and tool for how to carry out a successful waste management, meeting with goals and expectations. With time, it has also resulted in a closer and efficient interaction with other plans in the municipalities and in the cooperation between different stakeholders and authorities in the municipalities, aiming for sustainability.

### Producers responsibility

In 1994, the Government decided upon an Enactment on Producer’s Responsibility for packaging and wastepaper, after 3–4 years of very intensive discussions and debates between the Government, the Swedish EPA, The Swedish Federation of Municipalities, The Swedish Confederation of Industry and Avfall Sverige. They all agreed that there was a need for a producer’s responsibility with the economic responsibility on the producers, but they could not agree upon who should be responsible to carry out the actual collection of the packaging and the wastepaper from the households. Avfall Sverige argued for one judicial body, the municipalities, responsible for the collection of household waste *as well as* the collection of packaging and wastepaper from the households. The municipalities were already responsible since 1972 for the collection of household waste and could without practical problems and without any confusion for the households also, by themselves or by private contractors engaged with the municipalities, carry out the collection of packaging and wastepaper. However, the Government finally decided upon a split responsibility, where household waste was collected by the municipalities and the packaging by the producers or by them engaged with private contractors. During more than 25 years that split responsibility caused uncertainty in the households about who was responsible for what, uncertainty about who was responsible for information and services to the household and problems if it was the municipality or the producers that was responsible for taking care of the littering problem at and around the collection points/the recycling stations. With time, many municipalities had also the intent to offer households an increased service with collection close to the property of *all* different waste fractions, including packaging and wastepaper.

After more than 25 years of discussions and criticism, the Swedish Government 2022 decided on new rules for the collection of packaging from households and business activities, with the aim to facilitate for the producers to increase recycling and to achieve a more resource-efficient waste management (SFS, 2022). The aim is also to reduce the need for new virgin materials, as well as the emissions of carbon dioxide associated with their production.

The new rules put an economic responsibility on the producers to finance an efficient collection of the packaging. The municipalities are given the responsibility for the collection of packaging from households and from recycling stations in public places. The municipalities will be economically reimbursed by the producers for the work they carry out. The new rules will come into force on 1st of January 2024. Later on, from1st of January 2027, collection of packaging from the households, close to the property will be implemented, most likely in many places with the system described above, with two bins, four boxes in each for source separation of eight different recyclable fractions will be included for packaging materials.

### Hazardous waste and electronic scrap

In 1975, the Swedish municipalities were given a possibility by the law to voluntarily extend their responsibility to also include the collection of hazardous waste from the industry. The collection of hazardous waste from households was already included in the Municipal Waste Management Act in 1972 (SFS, 1970). At the end of the 1970s, some regions and municipalities had already extended the collection of hazardous waste to include also hazardous waste from the industry, and even more municipalities did so in the 1980s.

In 1985, there was a Governmental Ordinance on Hazardous Waste (SFS, 1985) and in 1989 an Ordinance on Hazardous Batteries (Cd, Hg, Pb) (SFS, 1989). The use of these elements in products became outlawed in principle, but in practice, only a marginal difference in the distribution of these elements was seen at first.

The service and the collection, where there was an extended municipal responsibility, worked so well that the Government decided upon a mandatory responsibility for hazardous waste from the industry for all municipalities in Sweden. That mandatory responsibility lasted for some years. In the 1990s, the Government changed it back to a voluntary responsibility as the industry, and the producers were expected to handle the hazardous waste themselves, as they had proved to be able to do in a satisfying way. In 2007, the possibility for the municipalities to voluntarily extend their responsibility to include even hazardous waste from the industry ceased.

Different systems for the collection of household hazardous waste were by time developed in different parts of Sweden, with the possibility for the public to deliver among other batteries, small-sized electronic scrap Waste from Electrical and Electronic Equipment (WEEE) and light bulbs in special boxes and containers at shopping centres, at recycling centres, etc.

In 2001, a Swedish nationwide compliance scheme for consumer and business-to-business (B2B) products was founded. It was initiated by 20 trade associations as a not-for-profit service organisation to serve all importers and manufacturers obliged by extended producer responsibility to take back electrical and electronic products.

The safe and correct handling of household hazardous waste and of electric and electronic scrap would not have been possible without the households’ participation in an efficient source separation, and responsible participation from engaged producers.

### Sweden joins European Union

In 1995, Sweden became a member of the European Union (EU). Eager to live up to all agreements, the Swedish waste management industry has applied the regulations developed within the union, often with some margin. This also means that the development of Swedish waste management resembles that of other European countries more and more. However, there are still a few factors that make Sweden stand out from the average EU member state.

One factor is the strong integration of waste treatment in the energy sector in a great part due to the existence of district heating systems that facilitate a good energy yield. For the last decades, the waste incineration capacity of Sweden has become more and more excessive in relation to domestic waste fuel production, and there has been a growing dependency on the import of waste fuels.

Between 1995 and 2000, there are even more source separation of waste in households and in the industry, with increased collection and recycling of different materials. All over Sweden, a large number of Recycling Centres are established for households to visit by car, dropping bulky waste, garden waste, recyclables, burnable waste, hazardous waste and electrical and electronic scrap.

From 2000 and onwards, there are even more EU directives and EU regulations with even stricter environmental demands on emissions and even stricter demands of better use of the resources in different wastes. Directives and regulations, which the EU-member states, have to meet with and harmonize with.

### Tax on WtE

In Sweden, there is a tax on landfilling from 2000 onwards. In 2002, there is a national ban on landfilling of burnable waste and in 2005 on organic waste. All of course resulting in a significant reduction of the amount of waste being landfilled.

Between 2006 and 2010, there was a tax on WtE, based on the amount of fossil content of the waste being incinerated. The tax ceased in 2010 when a governmental report stated that the tax did not result as originally expected in less incineration and more recycling, and only increased the costs of waste management for the households. The tax on WtE came back in 2020 and ceased once again from the 1st of January 2023, with the same reason as in 2010; the tax does not result in any incentive for increased source separation and recycling. Will the tax come back a third time? You can never tell!

### Food waste is a resource

Source separation in the households in the early 2000s turned out to be a very successful way of separating different recyclables, but there was still a need of taking care of the food waste in a better way and as a resource. Separate collection of food waste, from households, restaurants and similar places started already in 2003 and increases all the time. In 2003, the first goal set up by the Swedish government was to collect 35% by 2010. In 2020, 40% of the food waste was treated biologically for recovery of nutrition. Food waste is collected and delivered to biogasification plants with the production of a bio-fertilizer, to be used as a soil improver, and the production of biogas, mainly to be used as a vehicle fuel. Food waste or a slurry of food waste is today delivered to an increasing number of biogas plants.

Latest by the end of December 2023, there is an obligation for all Swedish municipalities to have systems installed for source separation and collection of food waste. The Government has declared that by the end of 2023, at least 75% of the food waste from households, commercial kitchens, restaurants and shops will be source-separated for biological treatment, with a production of nutrition and biogas. A very tough challenge! Will it be possible?

### Old clothes and textiles

For a long time, there has been a growing awareness in Sweden that discarded clothes and textiles have to be taken care of, reused and recycled in a much more sustainable way. The collection of old clothes and textiles increases, for reuse and for recycling, with the ambition to manage to separate them on different qualities, on different colours, with a quality secured declaration on the content of different materials, on different fibres, etc. However, there are difficulties in achieving a satisfying quality separation and marketing of reused and recycled clothes and textiles. Too many clothes and fibres are rejected for quality reasons and end up in waste incineration or in a landfill.

A breakthrough maybe the mechanical sorting facility Siptex, at Sysav, Malmö, Sweden. The only large-scale sorting facility of its kind on textiles and fibres, which so far meets and fulfils all the criteria for successful marketing and recycling (Sysav, 2022).

Much better cooperation between the different stakeholders is needed, especially including the producers.

In May 2023, the Swedish Government presented a memorandum with a proposal for even more source separation – a mandatory source separation all over Sweden for old clothes and textiles to be in force from 1 January 2025, handled within an extended municipal responsibility (Government, 2023).

### Recycling, energy recovery and landfilling in 2022

**Figure 5.**
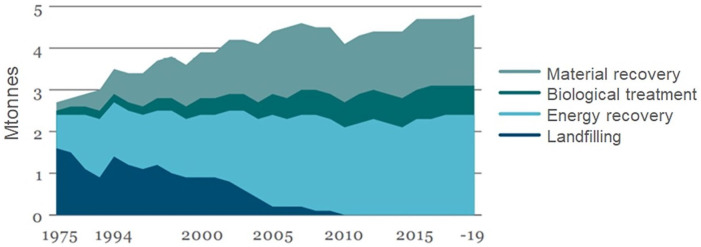
Treatment of household waste in Sweden from 1975 to 2019.

In 2022, there was a total collection of 4.7 million tonnes of household waste in Sweden, corresponding to 449 kg per person and year, a decrease of 5% compared with 2021. Is the decrease a trend of a future change or is it just a result of less consumption during the pandemic? The material recycling was estimated to be 28%, 16% was treated biologically for the production of manure and biofuel, 55% was treated in WtE-plants for the production of heat and power and only about 1% was sent directly to landfilling, mostly inert materials. (Avfall Sverige, 2023). In 2022, there were 36 landfills, many owned and operated by regional municipal waste management companies, compared with 1500 dumpsites/badly operated landfills at the beginning of the 1970s. In 2022, each person in Sweden produced 460 kg of household waste annually, compared with 200–250 kg in 1970 (Figure 5).

## Development of Swedish waste research

Fu et al. (2010) performed a global bibliometric study on journal articles dealing with waste published between 1993 and 2008; they saw a steady increase over the period and their findings predicted that the number of publications in 2013 would be twice that of what was published in 2008. About the same development occurred in the Swedish research output, and the number of theses in the area is perhaps an even better measure of the research interest.

**Figure 6.**
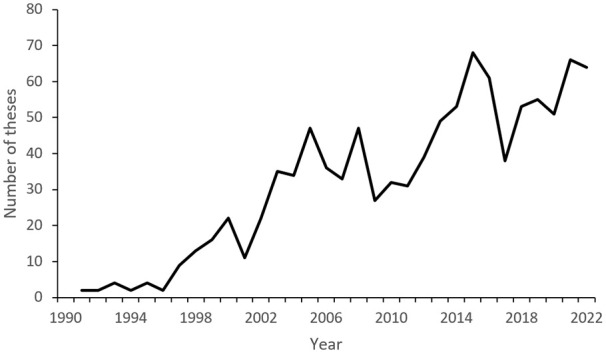
The distribution of PhD-theses dealing with waste issues between 1990 and 2022.

Swedish university publications are indexed in the open-access Swedish (university) publications (SWEPUB) (http://www.swepub.kb.se/) database. Searching for the term *waste* in combination with different aspects of waste management will generate a list of about 1000 PhD-theses up until and including 2022, only a few of them before 1990 (see Figure 6). However, many theses that are just remotely connected to waste management will be on the list, and at the same time, a number of theses that should have been there will be missing. This may be typical for a young and developing academic field where the terminology changes over time and overlaps with adjacent fields. So, the number is probably a somewhat inflated estimate of the academic productivity in the waste management area, but still likely in the right order of magnitude.

A 1000 theses correspond to 3000 years of research work, a fair amount of time, so one may wonder what has been done, why it was done and who did it. Another issue is if it has had much impact on waste management practices.

A little more than half of the theses defended before 2023 were done so in engineering disciplines, natural science follows with almost a third and the residual almost 20% are distributed among social science, agricultural science, medical and health sciences and humanities. The interest in waste research seems to diffuse into new areas of research with time. Looking at institutions, one may note that five universities account for more than 70% of the hits, while the remaining are distributed over 22 universities. Thus, it seems that the research interest is well routed in some organizations, although there are still few examples of disciplines defined in the area of waste research. One exception is the Chair of Waste Science and Technology discipline of Luleå University of Technology, which was established in 1980. Looking at the drivers of research, one needs to know that most of the academic research in Sweden is funded through national agencies that will define problem areas of interest. So, the topics researched will be influenced by what is perceived as relevant on a national political level. Also, environmental legislation impacts directly on both the focus of practitioners and, indirectly on the research that is funded. This policy orientation is also common in EU research funding.

As an illustration of this top-down influence on research funding, one can point to the research on landfill gas. In the decades following the so-called energy crises of the seventies, a fairly large volume of research went into developing control techniques and the use of landfill gas, globally and in Sweden. Among the studies performed in Sweden was one financed by the Swedish Energy Authority named ‘Samordnad deponigas-FUD’ (Coordinated landfill gas, research, development and demonstration). In this study, about 0.1 Mton of MSW was used in test cell experiments, at the time corresponding to about 10% of the total landfilled amount of this waste stream nationwide. Several theses were generated through this project alone.

But following the ban on landfilling of organic material in 2005, not a single thesis has been published on this theme. An ironic observer may wonder if the millions of tonnes of landfilled organics and the associated problems vanished.

According to a survey covering the years 1994–2003 (Lagerkvist, 2006), there were about ten theses published per year indicating a limited academic research activity in relation to the internal R&D of the waste management industry in the same period. It was also observed that:

– The interaction between industry and academia was often lacking.
– Waste research was gaining in quantity and diversion over time.
– Much of the early work seemed to be motivated by, and a reaction to, changing environmental protection legislation. However, towards the end of the period, some theses were done that seemed to be motivated more by a proactive development interest from within the industry.

**Figure 7.**
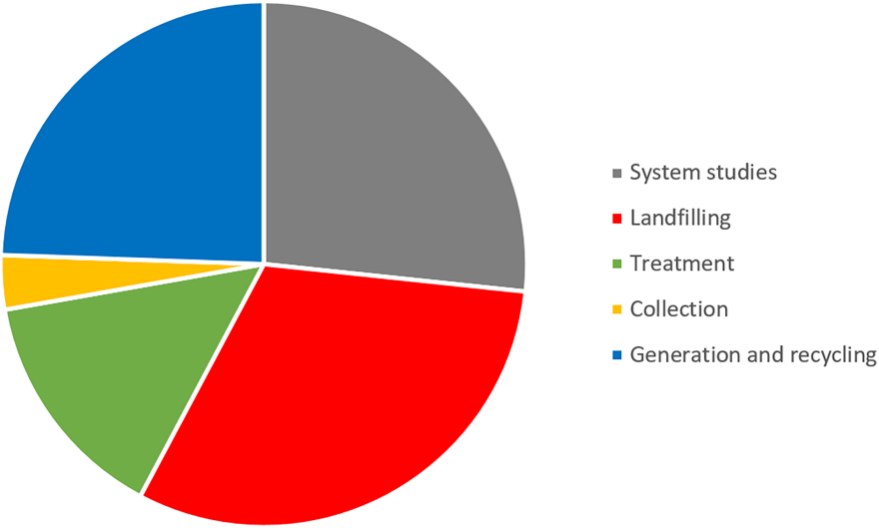
Academic theses 1994–2003 and their themes. Modified after Lagerkvist (2006).

The first observation was illustrated by the lack of PhD-theses dealing with waste collection, see Figure 7, during a period when this was a main concern for the waste management industry. The number of theses dealing with landfill issues, often leachate treatment, can be connected to the development of environmental legislation, mainly such that increased the demands on the industry concerning emission control. The large proportion of system analyses could also be seen as an indication of how new disciplines started to have an interest in waste management in the period since it is an easier and quicker way of acquiring a basic understanding of an area than the generation of original data is. At the time, such studies were more in demand by environmental authorities, trying to predict and evaluate the impact of policy changes. Contrasting to that, the number of theses dealing with waste treatment, for example, incineration or biological treatment, is a surprisingly low fraction of the total number of theses, given the large investments in treatment facilities during the period, and the corresponding internal research and development initiatives within the industry.

**Figure 8.**
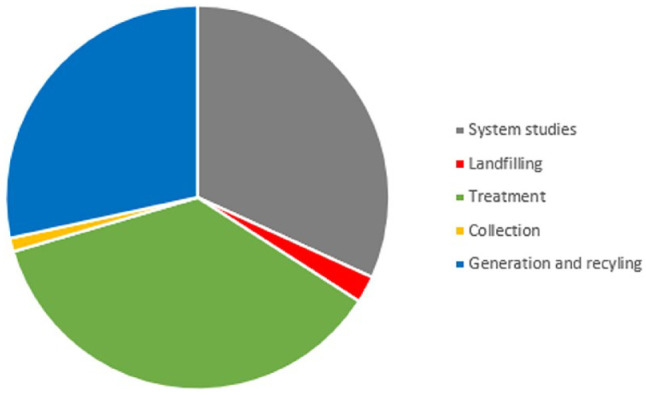
Academic theses 2010–2015 and their themes (Lagerkvist, 2016).

A follow-up study was made in 2016, and it was then concluded that there was an increase in academic production, and using the same classification as above, the distribution of theses is shown in Figure 8.

With reservation about the precision in classification, it seems like the interest among academics in waste collection systems remained limited, and the interest in system analysis stayed large, as it also was towards the end of the previous period. The interest in landfill research plummeted, and the interest in recycling and treatment increased, especially the interest in biological treatment. Another difference between the studied periods is that municipal solid waste was the main focus in the earlier period, but in the later period, also industrial waste problems received more attention. One can notice as well that generic trends like the aspect of greenhouse gas emissions become more like a standard item in problem descriptions and system evaluations with time.

Looking in a similar fashion to what has been published after 2015, the trends seem to hold, the number of theses seems to have stabilized around 50 per year and their themes seem to be similar to the 2010–2015 period. Industry involvement seems to increase, perhaps due to an increasing number of employees with research experience. Looking at the wider academic environment, almost 3000 PhD-theses are produced annually in Sweden, judging by the terms used in the searches. Almost two percent of the PhD students have expressed an interest in waste issues.

## Lessons learned and outlook

Looking back at the developments of Swedish waste management, one can conclude that it is not a linear nor a consistent process. Many external factors will influence what can be done and how it will be done. Public perception and political trends are influential, as is also the development of technology and the economy.

As a case in point, one can regard the once commonplace source grouping of different waste fractions, such as kitchen waste, nightsoil and ashes, and their subsequent use. With the emerging global trade of the 20th century, and innovations like canned food and the waste chute in apartment buildings, the materials got more mixed and harder to use. At the same time, strong growth in the economy and consumption potential made recycling seem less important. Towards the end of the last century, and only after a detour through a grooving concern of the waste’s detrimental impact on the environmental quality and a sudden hike in oil prices, then the resource interest saw a renaissance.

So, the challenge for waste management is always to keep up with changing external factors, and change can come fast sometimes. However, the basic mission of waste management, that is, to manage waste in an efficient way, and protect human health and environmental quality, will remain, and constitutes a service of fundamental importance to society.

Most people have little knowledge of waste management and its conditions, and this is probably why you can see unrealistic expectations expressed sometimes, for example, the idea of a future without waste. The waste management industry must have a voice in the public discourse, actively help to spread relevant information and explain that the laws of thermodynamics also apply today.

Circular economy is a concept that can invite such misunderstandings. It is often presented with a circle that just before completing a lap leaves a trickle of a stream called waste. The image is there to motivate, but it is too simplified, and it obscures the fact that waste will be generated in every step of a materials journey through society. Better information is needed, and the waste industry can provide it.

One factor that can be of help is new information technologies. Already today many sensors are used, for example, to schedule pick-ups, also weighing bins and image analyses are contributing to a wealth of information that was not previously available. Here, the issue of personal integrity may get more focus in the future.

Regarding waste research, it is even more exposed to external trends than the industry, due to the funding by grants from organizations that develop different ideas and programmes for the research they judge to be needed. A way to create a better continuity of waste research would be to create more active cooperation between industry and research institutions. Due to the build-up of internal R&D competence in the waste management industry, a better understanding of the role and conditions of research has gradually developed over the last 50 years, so what remains is to make more researchers understand the waste management industry better, and then both communities will get benefit. One could, for example, use the instrument of research schools organized jointly by industry and academy.

Looking to the near future, source separation has turned out to be a very successful tool, and it will be increasingly applied. As well known, a high collection rate of recyclables is not always synonymous with a high recycling rate. Today, most of the collected materials are recycled to a certain extent, but for the plastics. Of all plastic waste from the households being collected only about 15% is recycled, the rest is delivered to the Swedish WtE plants. Indeed, with energy recovery, but with a fossil content. With more other materials being recycled and not delivered to the WtE plants, the fossil content in the burnable waste will increase and has already increased the emissions of carbon dioxide significantly. As a reaction to this, several Carbon Capture and Storage/CCS projects are now underway. The cost for a CCS is extremely high, and it will take a considerable time before it is fully developed and operationally implemented. This problem cannot be solved by waste management alone, the producers also need to be involved to develop rational recycling options. However, should the material recovery of plastic become successful, then the shortage of waste fuel in Sweden, would become even more profound.

Regarding biological waste treatment, an EU decision recently abolished the Swedish tax exemption for biomethane. This may lower the interest in anaerobic waste treatment in the coming years, and perhaps lead to a diminishing number of plants, when the smaller and less profitable ones may close.

The diminishing flow of MSW to landfills has been replaced with large volumes of contaminated soils after an authority decision of remediation action has been taken. Thus, one may argue, as with the incineration tax, that the tax has lost its rationale of existence since it does not influence the recycling rates anymore, but it does generate a steady income for the state.

The waste management community of Sweden is doing well today and will probably continue to do so in the future. Over the last 50 years, capacity and competence have increased tremendously, and one may now see a tendency to develop more cooperation with other economic sectors and society at large to further the goals of waste management.
